# Novel Pegylated Interferon for the Treatment of Chronic Viral Hepatitis

**DOI:** 10.3390/v14061128

**Published:** 2022-05-25

**Authors:** Yi-Wen Huang, Albert Qin, Chan-Yen Tsai, Pei-Jer Chen

**Affiliations:** 1 Division of Gastroenterology and Hepatology, Department of Internal Medicine and Clinical Research Center, Taipei Medical University Hospital, Taipei 110, Taiwan; yiwenhuang@tmu.edu.tw; 2 Division of Gastroenterology and Hepatology, Department of Internal Medicine, School of Medicine, College of Medicine, Taipei Medical University, Taipei 110, Taiwan; 3 School of Medicine, National Taiwan University College of Medicine, Taipei 100, Taiwan; 4 PharmaEssentia Corporation, Taipei 115, Taiwan; albert_qin@pharmaessentia.com (A.Q.); chanyen_tsai@pharmaessentia.com (C.-Y.T.); 5 Graduate Institute of Clinical Medicine, National Taiwan University College of Medicine, Taipei 100, Taiwan; 6 Hepatitis Research Center, National Taiwan University Hospital, Taipei 100, Taiwan

**Keywords:** clinical trial, chronic hepatitis B, chronic hepatitis C, chronic hepatitis D, interferon, ropeginterferon alfa-2b

## Abstract

Ropeginterferon alfa-2b is a novel mono-pegylated and extra-long-acting interferon, being developed for the treatment of myeloproliferative neoplasm (MPN) and chronic viral hepatitis. It has a favorable pharmacokinetic profile and less frequent dosing schedule, i.e., once every two to four weeks, compared to conventional pegylated interferon products, which have multiple isomers and are administered weekly. It was approved for the long-term treatment of polycythemia vera, an MPN, and has been included in the NCCN clinical practice guidelines for this indication. Ropeginterferon alfa-2b has demonstrated efficacy and showed a favorable safety profile for the treatment of chronic viral hepatitis in several clinical studies. In this article, we review its pharmacokinetics and available clinical data and suggest that ropeginterferon alfa-2b administered once every two weeks can serve as a new treatment option for patients with chronic viral hepatitis, including chronic hepatitis B, C, and D.

## 1. Introduction

Ropeginterferon alfa-2b is a novel mono-pegylated proline-interferon (pro-IFN) alfa-2b. It is predominantly a single and chemically homogenous isomer, as compared to the conventional pegylated interferon (IFN) products, which contain multiple isomers that may contribute to the development of adverse effects [1,2]. Ropeginterferon alfa-2b has favorable pharmacokinetic properties that allow it to be dosed on a much less frequent schedule, i.e., once every 2 weeks or every 3 to 4 weeks [3,4,5]. The drug has been shown to be well tolerated and efficacious in patients with polycythemia vera (PV) and has been approved as the first IFN-based therapy for the PV treatment in Europe, Switzerland, Israel, Taiwan, Korea, and the USA [6,7,8]. It has recently been included in the NCCN clinical practice guidelines [9].

Another major application of IFN alfa (alpha) is for the treatment of chronic viral hepatitis, such as hepatitis B, C, and D. To identify the clinical values of ropeginterferon alfa-2b in these clinical indications, ropeginterferon alfa-2b has been evaluated in phase 1 and 2 clinical trials for the treatment of chronic hepatitis B (CHB) and chronic hepatitis C (CHC) [10,11,12]. We summarized the relevant clinical results to show that ropeginterferon alfa-2b as a novel IFN alfa-based therapy was well tolerated and effective in these clinical studies and discuss the future perspectives in using ropeginterferon alfa-2b for the treatment of chronic viral hepatitis.

## 2. Pharmacokinetics (PK) and Pharmacodynamics (PD) of Ropeginterferon alfa-2b in Healthy Volunteers

The PK/PD profiles of ropeginterferon alfa-2b were evaluated in three clinical studies of healthy volunteers, including one first-in-human study conducted in Canada, one in China, and another in Australia with Japanese and Caucasian subjects [3,4,5]. Ropeginterferon alfa-2b was subcutaneously (SC) administered as a single injection at doses ranging from 24 to 300 μg in these studies (Table 1). The T_max_ of ropeginterferon alfa-2b was found to be 75 to 142 h, and the elimination half-life was 52 to 129 h. The non-dose proportionality of ropeginterferon alfa-2b was observed in all three studies. The slope of the relationship between the dose and the exposure variables, i.e., AUC_0-inf_, AUC_0-t_, and C_max_, were greater than one, suggesting that the increase in ropeginterferon alfa-2b exposure was greater than dose proportional under 300 μg in healthy subjects. 2′,5′-Oligoadenylate synthetase (OAS), neopterin, and β2-microglobulin were evaluated as PD markers in these studies. Overall, a dose-dependent increase for the serum concentration of the PD markers was observed.

Compared to the conventional pegylated interferon products, e.g., peginterferon alfa-2a, ropeginterferon alfa-2b had a higher drug exposure at the same dose level, i.e., 180 μg [3,4]. At the same 180 µg dose level, ropeginterferon alfa-2b showed higher C_max_, AUC_0-t_, and AUC_0-inf_ than peginterferon alfa-2a [3,4]. The geometric mean ratios of AUC_0-inf_, AUC_0-t_, and C_max_ were 1.66, 1.82, and 1.76, respectively, for ropeginterferon alfa-2b when compared to peginterferon alfa-2a [3]. The C_max_ of ropeginterferon alfa-2b was 20.7 ng/mL as compared to 12.95 ng/mL of peginterferon alfa-2a [3]. The C_max_ was reached in a shorter time (T_max_) in the ropeginterferon alfa-2b than in the peginterferon alfa-2a group: 79.22 h vs. 84.25 h [3]. The half-lives (T½) of ropeginterferon alfa-2b and peginterferon alfa-2a were 66.53 and 89.32 h, respectively [3].

Ropeginterferon alfa-2b at a single administration showed similar PD profiles in the OAS and neopterin bioactivity as peginterferon alfa-2a [3]. At the single dose, the safety profile of the ropeginterferon alfa-2b did not show a significant difference from that of peginterferon alfa-2a. Most of the adverse events were mild or moderate [3,4,5]. The phase 1 data indicated higher PK exposures and similar safety profiles of ropeginterferon alfa-2b at a single dose when compared to peginterferon alfa-2a in healthy volunteers and suggested that ropeginterferon alfa-2b could potentially be administered with less frequent injections in clinical use. The PK and PD parameters of ropeginterferon alfa-2b in these studies are summarized in Table 1.

## 3. Clinical Studies in Patients with Chronic Hepatitis

### 3.1. Ropeginterferon alfa-2b in Chronic Hepatitis C (CHC)

CHC infection causes inflammation of the liver, leading to a high risk of liver fibrosis, cirrhosis, liver failure, and hepatocellular carcinoma (HCC) [13,14]. CHC affects an estimated 71 million people worldwide and elimination of CHC has been advocated by the WHO as a continuous development goal till 2030 [15]. There are seven major genotypes of hepatitis C virus (HCV). Genotype 1 is the predominant (46%) followed by genotype 3 (22%) and genotype 2 (13%), and the prevalence of HCV genotypes 4, 5, 6, and 7 varies greatly with geographic locations [16,17]. In Asia, the prevalence of genotype 2 can be as high as 60% in certain areas [15,17].

IFN alfa therapy has been shown to be effective for the CHC and was widely used for the CHC treatment in the pre-direct acting antiviral agent (DAA) era [18]. IFN alfa binds to a heterodimeric transmembrane receptor termed IFN-α/β receptor (IFNAR) to activate the JAK-STAT signal transduction pathways and elicit their biological activities, including the inhibition of virus replication and activation of the immune responses [19,20].

Pegylated IFN alfa therapies have been shown to induce clinically appreciable sustained virologic responses (SVRs) in CHC patients, e.g., a 70–90% SVR rate in patients with the genotype 2 infection [21,22,23]. In addition, they also alleviate fibrosis and reduce the risk of HCC development in CHC patients [24,25,26]. To date, two pegylated IFNs, i.e., peginterferon alfa-2a and peginterferon alfa-2b, have been approved and clinically used for the CHC treatment [27,28]. However, their relatively frequent dosing schedule, i.e., once every week, and notable side effects such as flu-like symptoms, injection site reactions, and depression have limited their use in CHC treatment. By contrast, ropeginterferon alfa-2b showed a favorable dosing schedule and safety profile in the treatment of chronic hepatitis including CHC and CHB. Two phase 2 clinical studies have been conducted to explore and validate the safety and efficacy of ropeginterferon alfa-2b in combination with ribavirin in the CHC treatment, including one in CHC genotype 1 and the other in CHC genotype 2 (Table 2) [10,11].

In the phase 2 CHC genotype 1 study [10], 106 treatment naive patients were enrolled and randomized into four treatment groups including peginterferon alfa-2a once every week SC at the dose of 180 µg (Group 1) as a control, ropeginterferon alfa-2b SC once every week at 180 or 270 µg (Group 2 and Group 3, respectively) or once every two weeks at 450 µg (Group 4), plus daily oral ribavirin for a treatment of 48 weeks [10]. Patients who were treated with ropeginterferon alfa-2b at 180 µg showed a notably higher serum exposure (AUC_0-τ_), which was 1.4-fold greater than those treated with peginterferon alfa-2a at the same dose. The accumulation ratio of ropeginterferon alfa-2b in its 180 µg group was twofold greater than that of peginterferon alfa-2a at the same dose. The ropeginterferon alfa-2b groups had the SVR24 rates ranging from 66% to 80%, which were similar to that of the 180 µg peginterferon alfa-2a group (Table 2). Moreover, the ropeginterferon alfa-2b groups showed a favorable safety profile regarding flu-like symptoms, anxiety, and depression, when compared to the peginterferon alfa-2a group. The incidence of flu-like symptoms was 66.7%, 53.3%, 55.0%, and 48.3%; anxiety was 14.8%, 6.7%, 0% and 0%; and depression was 25.9%, 13.3%, 0%, 3.4% for the 180 µg peginterferon alfa-2a, and 180, 270, 450 µg ropeginterferon alfa-2b groups, respectively. Grade 2 or 3 depression was reported in the peginterferon alfa-2a group, but none in the ropeginterferon groups. Ropeginterferon alfa-2b administered once every two weeks at the dose of 450 µg showed a favorable safety profile regarding key adverse effects reported with previous IFN therapies compared to the weekly given, conventional pegylated IFN alfa-2a.

In another phase 2 study [11], 86 treatment-naive patients with CHC genotype 2 were randomized to receive daily oral ribavirin plus peginterferon alfa-2a weekly at 180 µg, or ropeginterferon alfa-2b once every two weeks at 270, 360, or 450 μg [11]. In this study, patients who achieved the rapid virologic response (RVR, undetectable HCV RNA at 4 weeks of treatment) received a total treatment of 16 weeks, while those without RVR received a total treatment of 24 weeks. At 12 weeks of treatment, the rate of 100% in the complete early virologic response (cEVR) was achieved in all groups. The end of treatment virologic response (ETVR), defined as undetectable HCV RNA at the end of treatment per protocol (i.e., 16 weeks or 24 weeks), was also achieved in 100% in all groups, except one patient who discontinued treatment early due to an AE at week 13 in the 360 μg ropeginterferon alfa-2b group (ETVR: 95.2%; 20/21; 95% CI: 76.2–99.9%). In this study, the SVR12 rates at 12 weeks of follow-up were observed to be 95.5%, 82.6%, 85.7%, and 70.0% and the SVR24 rates were observed to be 95.5%, 78.3%, 85.7%, and 60% in the 180 µg peginterferon alfa-2a weekly, 270, 360, and 450 µg ropeginterferon alfa-2b bi-weekly groups, respectively. The relapse rate was higher in the ropeginterferon alfa-2b groups when compared to the peginterferon alfa-2a group. Further analysis of the relapse rates in patients receiving 16- or 24-week regimens indicated that the 16-week treatment regimen, despite having impressive RVR and ETVR, may not be sufficient in achieving a high SVR24 rate and may have contributed to the higher relapse rate in the ropeginterferon alfa-2b groups. Consistently, the relapse rate in the ropeginterferon alfa-2b groups was lower than the peginterferon alfa-2a group in patients who received the 24 weeks of treatment (25% vs. 33%). Therefore, the completion of the 24-week treatment appears to be required for higher SVR rates. For safety, the injection site reactions were only noted in the peginterferon alfa-2a group but not in the ropeginterferon alfa-2b groups, which may be due to the less frequent dosing regimen of ropeginterferon alfa-2b, i.e., once every two weeks.

### 3.2. Ropeginterferon alfa-2b in Chronic Hepatitis B (CHB)

CHB is another public health problem worldwide. The WHO estimates that CHB affects 296 million people worldwide in 2019, with 1.5 million new infections each year [29]. It resulted in an estimated 820,000 deaths, mostly due to cirrhosis and HCC. Although CHB could largely be prevented by vaccines, it is hardly cured completely once infection occurs. The persistent presence of the covalently closed circular DNA (cccDNA) of the hepatitis B virus (HBV), which has a long half-life, is a major reason [30,31]. Pegylated IFNs and nucleos(t)ide analogues (NAs) including entecavir and tenofovir were approved and serve as the preferred first-line treatment for chronic HBV infection. For NAs, drug resistance can occur and is a critical factor in determining the success of the NA-based anti-HBV therapy. Entecavir and tenofovir are associated with high barriers to antiviral drug resistance compared to other NAs, but they achieve a relatively low HBeAg seroconversion rate of about 10–21%, and approximately 1–3% in the rate of HBsAg loss, which was regarded as a functional cure for the HBV infection [32]. The pegylated IFN alfa-based therapy showed higher seroconversion rates of hepatitis B e-antigen (HBeAg) (~30%) and surface antigen (HBsAg) (~3%) [32] and has a minimal risk of drug resistance. By immune stimulations, it can potentially cause the clearance of the viral genome. However, the long duration of treatment with a frequent injection schedule, i.e., once every week, and side effects could hamper therapy compliance and limit its use in the CHB treatment.

Ropeginterferon alfa-2b, with a convenient, infrequent dosing scheme and favorable safety profile, may potentially serve as a new treatment option for CHB. In a phase 1/2 study, 31 HBeAg-positive and 31 HBeAg-negative CHB patients were randomized to receive ropeginterferon alfa-2b once every two weeks at 350 μg (Group 1) or 450 μg (Group 2), or the conventional pegylated IFN alfa-2a (peginterferon alfa-2a) once every week at 180 μg (Group 3) [12]. Patients received 48 weeks of treatment with a follow-up of 24 weeks. At the follow-up week 24 (FW24), both the 350 and 450 μg ropeginterferon alfa-2b groups showed a higher cumulative HBeAg seroconversion rate than the peginterferon alfa-2a group: 27.3%, 36.4%, and 11.1%, respectively. The time to achieve HBeAg seroconversion was also shorter in the ropeginterferon alfa-2b groups than in the peginterferon alfa-2a group. The HBeAg seroconversion started in TW24, TW16, and TW48 in the 350 and 450 μg ropeginterferon alfa-2b groups and the peginterferon alfa-2a group, respectively. Ropeginterferon alfa-2b was well tolerated in the study. Most adverse events were mild or moderate. Depression was not observed in the study. In addition, the ropeginterferon alfa-2b groups showed a lower incidence of rash (9.5% and 4.5%, respectively) as compared to the peginterferon alfa-2a group (36.8%). Other study drug-related adverse events were comparable among treatment groups. This study showed that ropeginterferon alfa-2b once every two weeks was well tolerated and effective for the CHB treatment, with a higher HBeAg seroconversion rate than peginterferon alfa-2a once every week.

## 4. Future Perspectives for Ropeginterferon alfa-2b in the Chronic Viral Hepatitis Treatment

The clinical use of pegylated IFNs for the chronic hepatitis treatment has been limited in the past due to the need of frequent dosing and obvious side effects including injection site reactions, flu-like syndromes, and depression [27,28]. Ropeginterferon alfa-2b as a novel site-selective, mono-pegylated IFN alfa-2b has improved PK properties, meaning that is can be given on a less frequent schedule. This advantage, together with its favorable safety profile and significant clinical response, supports its use and further development for chronic hepatitis treatment.

Flu-like symptoms, administration-site reactions, and depression are the major concerns for previous IFN-based therapies. These side effects have been observed notably less often with the ropeginterferon alfa-2b treatment in several studies. This is possibly due to the fact that ropeginterferon alfa-2b is predominantly a single isomer and is administered on a less frequent schedule. Consistent with the data in the chronic hepatitis studies, the rate of depression from the long-term ropeginterferon alfa-2b treatment up to 5 years in the phase 3 studies in PV patients was also minimal [6]. This reinforces the argument for using ropeginterferon alfa-2b in the chronic viral hepatitis treatment.

DAAs improved SVRs for CHC patients. Treatment failures due to drug resistance or side effects still occur in a small portion of treated patients [33,34]. The reasons for DAA failures are heterogeneous, including the resistance-associated substitutions (RASs) that lead to viral variants with reduced susceptibilities to DAAs [33]. A new generation of DAA regimens with high rates of viral eradication above 90% regardless of the presence of RASs has been developed, e.g., voxilaprevir/velpatasvir/sofosbuvir. Their availability and the well-validated RVS testing may not be widely available, especially in the resource-limited area [35,36]. It was also reported that HCC could still recur in some patients who achieved the SVR after the treatment with DAAs [37,38,39,40]. Recent evidence with a larger number of patients shows that HCV clearance by the DAA treatment reduces the incidence of HCC [41]. Pegylated IFN alfa therapies could reduce the HCC risk by not only causing SVRs but also potentially eliciting IFN-associated intrinsic anti-cancer properties. Type I IFNs have known anti-cancer activities including the direct anti-proliferative effect consisting of cytotoxicity, cell cycle inhibition and induction of a senescence-like state, and the indirect anti-tumor effect induced by immune stimulation and anti-angiogenesis [20,42,43,44,45]. Type 1 IFNs alfa and beta bind a same receptor termed IFNAR to elicit their biological activities [19,20]. They selectively induce a cell-growth inhibitory effect such as cell-cycle inhibitions in human transformed or cancer cells, but not in normal cells [42]. Furthermore, low-copy gene delivery of IFN beta by a lentivirus vector or the extrachromosomal gene expression by an adenoviral vector led to the significant inhibition of tumor formation [44,46]. Recombinant IFN alfa was approved for the treatment of hairy cell leukemia, malignant melanoma, AIDS-related Kaposi’s sarcoma, and follicular non-Hodgkin’s lymphoma, and its pegylated products with extended half-lives were under development for the treatment of more cancer types, e.g., acute myeloid leukemia [47,48,49,50,51,52,53]. IFN-alfa therapies were also noted to significantly reduce the risk of CHC-associated HCC occurrence [26,54,55]. In the phase 3 studies in PV patients, there was no occurrence of secondary cancers in the patients who received ropeginterferon alfa-2b. In contrast, five events of cancer progression, including two cases of secondary acute leukemia, were observed in the control group [6,56]. Ropeginterferon alfa-2b was also found to be able to selectively inhibit the malignant JAK2 mutation-carrying progenitor cells in the bone marrow of patients [57]. Taken together, the data suggest that ropeginterferon alfa-2b is not only effective in chronic hepatitis treatment but also potentially inhibits cancer formation and growth. This further supports the use of ropeginterferon alfa-2b in the treatment of chronic viral hepatitis.

For CHC infection, ropeginterferon alfa-2b can be a viable option when DAAs are not appropriate such as in patients who had treatment failures due to drug resistance or side effects. Since they have different modes of action, a combination treatment with ropeginterferon alfa-2b and DAAs may potentially induce a combinatorial or complementary clinical effect in patients, being effective in curing CHC and decreasing the HCC risk. Therefore, with a less frequent dosing scheme, improved PK and safety profiles, and potential as an anti-cancer agent, ropeginterferon alfa-2b provides new treatment options for CHC patients.

Ropeginterferon alfa-2b can be very useful in the treatment of CHB and chronic hepatitis D (CHD). Pegylated IFN alfa was approved for the CHB treatment [28] and serves as a backbone treatment in CHD patients as an off-label use [58]. Pegylated IFN treatment can lead to higher rates of HBeAg and HBsAg seroconversion in patients, compared to those receiving NAs, possibly due to its immune stimulation or ability to enhance the cccDNA degradation and exert epigenetic modifications of the cccDNA transcription [59]. Ropeginterferon alfa-2b therapy has been shown to cause higher HBeAg seroconversion rates than the conventional pegylated IFN alfa-2a given weekly in the phase 2 setting [12]. Therefore, ropeginterferon alfa-2b is expected to be effective in CHB and CHD. NA therapies, especially their long-term treatment, are also effective in depleting the cccDNA [60]. However, there is a potential for a combination-therapy approach with NAs and a novel and effective pegylated IFN alfa such as ropeginterferon alfa-2b in curing CHB. It is also possible that ropeginterferon alfa-2b therapy may help CHB patients off the long-term NA treatment during their disease management.

There have been rekindled enthusiasms for combining small molecules with IFN-based therapies for the CHB or CHD treatment [61,62]. A meta-analysis indicated that the combination of the pegylated IFN alfa treatment with lamivudine given orally could show a better virological (57% vs. 20% in HBeAg-positive patients and 85% vs. 65% in HBeAg-negative patients, respectively) and biochemical (48% vs. 37% in HBeAg-positive patients and 50% vs. 40% in HBeAg-negative patients, respectively) responses than pegylated IFN alfa monotherapy at the end of treatment [63]. The pegylated IFN alfa treatment plus adefovir also had a better seroconversion rate than the pegylated IFN alfa alone in the HBeAg-positive patients (51% vs. 34%). In addition, higher rates of HBsAg loss (10% vs. 0%) and HBeAg seroconversion (29.5% vs. 15.6%) were observed in the combination treatment with pegylated IFN alfa and tenofovir than the tenofovir therapy alone [64]. The combination of the pegylated IFN alfa therapy with entecavir or tenofovir also led to a higher rate of HBsAg loss when compared to the NA treatment alone (30% vs. 7%) [65]. Furthermore, a reversion of hepatic fibrosis could be observed in the CHB patients who received the entecavir add-on pegylated IFN alfa therapy [66]. Patients treated with the combined therapies had a larger improvement of liver histology in the mean liver stiffness value when compared to the monotherapy. Finally, early combination of entecavir and the pegylated IFN alfa-2a treatment improved the anti-HBV efficacy and long-term survival in HBV DNA-positive HCC patients as compared to the NA monotherapy [67]. Previous combination studies with IFN-based therapies and NAs are summarized in Table 3. These data show that the combination regimens that contain a pegylated IFN therapy as a backbone treatment may cause a functional cure of CHB and deliver a therapeutic effect on HCC. Therefore, the availability of ropeginterferon alfa-2b as a new-generation pegylated IFN alfa might facilitate the development of new combination regimens in the search for the cure of CHB or CHD infections.

Approximately 5% of patients with CHB are co-infected with HDV, leading to the most severe form of chronic viral hepatitis [68,69]. HDV is a defective virus that requires coinfection with HBV to replicate. Envelope proteins from HBV, i.e., small-HBsAg (S-HBsAg), medium-HBsAg (M-HBsAg), and large-HBsAg (L-HBsAg), are essential for HDV to enter and replicate in the liver cells [70]. Compared to the HBV infection alone, HBV/HDV co-infection results in a threefold increase in the risk of liver cirrhosis, with approximately 70% of cases developing cirrhosis within 5 to 10 years of diagnosis [71]. It could also cause a higher risk of HCC development with an odds ratio of 1.28–2.77 when compared to HBV infection alone [72]. Until now, no medication has been approved by the FDA for the treatment of CHD. IFN-based therapies have long been recommended for the off-label treatment of CHD by both the American Association for the Study of Liver Diseases (AASLD) and European Association for the Study of the Liver (EASL) [73,74]. A 48-week treatment with pegylated IFN alfa given weekly showed an HDV RNA clearance rate of 20–30% in HDV patients [75]. Long-term follow-up studies indicated that the IFN alfa-based therapies led to a lower likelihood of liver-related complications in CHD patients [76,77,78]. However, late HDV RNA relapses may occur after the pegylated IFN alfa therapy [79]. New therapies such as inhibitors of viral cell entry or secretion and nucleic acid polymers (NAPs) were subsequently being developed for HDV treatment [69]. Bulevirtide is a synthetic lipopeptide which inhibits the entry of HDV and HBV into hepatocytes [69]. In 2020, bulevirtide at 2 mg SC once daily was conditionally approved in the EU for the CHD treatment [80]. A 24-week regimen with bulevirtide at 2 mg plus tenofovir showed a higher HDV RNA clearance rate at the end of treatment than tenofovir alone (53.6% vs. 3.6%) [81]. HDV viral RNA relapse was reported in approximately 40–70% of the HDV RNA responders who received bulevirtide plus tenofovir [82]. By contrast, a 48-week regimen containing 2 mg bulevirtide and pegylated IFN-alfa had a higher HDV RNA clearance rate (80%) than bulevirtide (13%) or pegylated IFN alfa alone (13%) at the end of treatment [81]. The proportion of patients with undetectable serum HDV RNA remained higher in the combination group than the pegylated IFN alfa treatment alone group (53% vs. 0%) [83]. Complementary effects for the HDV treatment were also reported in the combination of a pegylated IFN alfa therapy with other antiviral agents. Lonafarnib, an oral small-molecule drug which inhibits the prenylation of large hepatitis D antigen (L-HDAg), blocks the assembly and secretion of HDV [70]. A triple combination therapy of lonafarnib, pegylated IFN alfa, and ritonavir showed a significantly higher virological response rate than the dual combination therapy of lonafarnib plus ritonavir (89% vs. 46%) [84]. Combination therapy with pegylated IFN alfa and NAPs also showed great potential for clearing the HDV RNA. NAPs are phosphorothioated oligonucleotides and can inhibit HDV infection [85,86,87,88,89,90,91]. Adding NAPs to the pegylated IFN alfa-based backbone therapy showed greater HDV RNA clearance in patients with HBV/HDV co-infection [77,92]. These clinical data suggest that combination regimens containing a pegylated IFN alfa-based backbone therapy are effective for the treatment of HBV/HDV co-infection. As an improved site-selective pegylated IFN alfa product, ropeginterferon alfa-2b could potentially serve as a better backbone treatment in combination regimens for CHD patients.

Immunotherapy is an attractive area for developing effective CHB treatments. Functional dysregulation or exhaustion of CD8^+^ T cells occurs in patients with CHB [93]. PD-L1, the ligand of programmed cell death 1 (PD1), is present on liver cells but overexpressed during CHB infection [94]. Blockade of PD1/PD-L1 signaling, a targeted immune checkpoint inhibition, re-activated the specific T cell responses against HBV in animal models [95,96]. In a clinical study in patients with advanced HCC, CHB patients who received the anti-PD-1 antibody treatment once every two weeks at 3.0 mg/kg showed a one-log decline in the HBsAg level [97]. Significant HBsAg declines from baseline were also observed after a single dose of an anti-PD-1 blockade antibody [98]. These clinical studies suggest a potential of anti-PD-1 antibodies in the treatment of chronic HBV infection. It is worth noting that HBV reactivation was observed in HCC patients with HBV infection after immuno-checkpoint inhibition and that the reactivation resolved after anti-viral treatments [99,100,101]. Combination therapy with anti-PD1/PD-L1 antibodies and pegylated IFNs may help clear the viral genome and be effective in preventing the HBV reactivation. Type 1 IFNs can stimulate the innate and acquired immunities, including the direct activation of the CD8^+^ T cells without the CD4+ T helper cells [102], and can potentially augment the effect of the targeted immune checkpoint inhibition by the anti-PD1/PD-L1 antibodies. Therefore, a clinical study is being conducted to explore the efficacy and safety of the combined therapy of ropeginterferon alfa-2b and an anti-PD1 antibody in CHB and CHD patients (ClinicalTrials.gov No. NCT04638439). The combination approach of anti-PD1/PD-L1 and ropeginterferon alfa-2b therapies might offer a more effective treatment for the CHB.

## 5. Conclusions

Ropeginterferon alfa-2b has shown a favorable tolerability, safety, and efficacy profile in chronic viral hepatitis patients. By reducing the hurdles of using an IFN alfa-based therapy for the chronic viral hepatitis, it provides new treatment options for chronic viral hepatitis patients, especially in combination therapies with other anti-viral agents for CHB or CHD treatment.

## Figures and Tables

**Table 1:** PK/PD parameters of ropeginterferon alfa-2b in clinical studies of healthy volunteers.

Parameter	Healthy Subjects			
	A09-102 Study [3]	A17-101 Study [4]	A17-102 Study [5]	
Study population	Canadian	Chinese	Japanese	Caucasian
SC dosing	Single dose			
Dose range (µg)	24 to 270	90 to 270	100 to 300	
Sampling time (h) range	0 to 672	0 to 672	0 to 672	
**PK parameters**				
T_max_ (h) range *	75 to 116	92 to 142	108 to 111	84 to 108
t_1/2_ (h) range	61 to 118	78 to 129	67 to 69	52 to 112
C_max_ (ng/mL) range	1.8 to 24.8	4.2 to 24.1	8.4 to 41.4	4.5 to 19.2
AUC_0-t_ (ng·h/mL) range	273 to 6068	957 to 6983	1445 to 7658	945 to 3933
AUC_0-inf_ (ng·h/mL) range	372 to 6258	1287 to 7998	1927 to 7843	1510 to 5433
**Dose proportionality analysis**				
**ln (AUC_0-inf_)**				
Slope (95% CI)	1.22 (1.00–1.44)	1.84 (1.13–2.55)	1.35 (0.93–1.76)	1.11 (0.06–2.16)
**ln (AUC_0-t_)**				
Slope (95% CI)	1.36 (1.11–1.61)	2.52 (0.69–4.35)	1.73 (1.30–2.16)	1.52 (0.31–2.72)
**ln (C_max_)**				
Slope (95% CI)	1.19 (0.99–1.39)	1.87 (0.79–2.95)	1.52 (1.11–1.94)	1.49 (0.59–2.40)
**PD parameters**				
**2′,5′-Oligoadenylate synthetase**				
E_max_ (pmol/dL) range	266–568	NA	NA	NA
ET_max_ (h) range	160–222	NA	NA	NA
AUEC_0-t_ (h·pmol/dL) range	51,970–175,233	NA	NA	NA
**Neopterin**				
E_max_ (nmol/L) range	14–20	NA	19.70–42.87	26.06–40.50
ET_max_ (h) range *	48–104	NA	36–48	48
AUEC_0-t_ (h·nmol/L) range	1213–3328	NA	6298–11,690	8897–12,510
**β2-microglobulin**				
E_max_ (µg/mL) range	NA	3.126–3.341	2.356–3.252	2.681–3.644
ET_max_ (h) range *	NA	118–132	84–120	72–84
AUEC_0-t_ (h·µg/mL) range	NA	1608–1775	1207–1649	1341–1856

Abbreviation: AUC_0-inf_: area under the serum concentration-time curve from time zero to infinity; AUC_0-t_: area under the serum concentration–time curve from time zero to the last measurable concentration; AUEC_0-t_: area under the serum concentration–time curve PD biomarker from time zero to the last measurable concentration; CI: confidence interval; C_max_: Maximum serum concentration; E_max_: maximum serum PD biomarker response; ET_max_: the time that E_max_ was observed; ln: natural logarithm; SC: subcutaneous; t_½_: elimination half-life; T_max_: time at which C_max_ was observed. * For T_max_ and ET_max_, the data are expressed as mean value in A09-102 and A17-101 studies while expressed as median in A17-102 study. For the other PK and PD parrameters, i.e., t_½_, C_max_, AUC_0-t_, AUC_0-inf_, E_max,_ and AUEC_0-t_, the data are expressed as mean value in A09-102, A17-101 and A17-102 studies.

**Table 2:** Efficacy of ropeginterferon alfa-2b in CHC treatment.

Parameters	CHC Genotype 1				CHC Genotype 2			
	A11-201 Study [10]				A11-203 Study [11]			
	Peginterferon alfa-2a	Ropeginterferon alfa-2b			Peginterferon alfa-2a	Ropeginterferon alfa-2b		
	180 µg (Group 1) *n* = 27	180 µg (Group 2) *n* = 30	270 µg (Group 3) *n* = 20	450 µg (Group 4) *n* = 29	180 µg (Group 1) *n* = 22	270 µg (Group 2) *n* = 23	360 µg (Group 3) *n* = 21	450 µg (Group 4) *n* = 20
**SVR12**	74.1%	70.0%	80.0%	69.0%	95.5%	82.6%	85.7%	70.0%
**SVR24**	77.8%	66.7%	80.0%	69.0%	95.5%	78.3%	85.7%	60.0%

Abbreviation: CHC: chronic hepatitis C; SVR12: sustained virologic response at 12 weeks post-treatment; SVR12: sustained virologic response at 24 weeks post-treatment.

**Table 3:** Combinations of PEG-IFN alfa and NA therapies in the CHB treatment.

Treatment Regimens	Study Type	Study Populations	Clinical Efficacy	References
PEG-IFN alfa + Lam vs. PEG-IFN alfa alone	MA	HBeAg-positive CHB	Virology response ^a^: 57% vs. 20% Biochemical response ^b^: 48% vs. 37%	[63]
		HBeAg-negative CHB	Virology response ^a^: 85% vs. 65% Biochemical response ^b^: 50% vs. 40%	
PEG-IFN alfa + ADV vs. PEG-IFN alfa alone	MA	HBeAg-positive CHB	HBeAg seroconversion ^c^: 51% vs. 34%	[63]
PEG-IFN alfa 2a + TDF vs. TDF alone vs. PEG-IFN alfa 2a alone	RCT	CHB	HBsAg loss ^d^: 10.4% vs. 0% vs. 3.5% HBeAg seroconversion ^e^: 29.5% vs. 15.6% vs. 25.0%	[64]
		HBeAg-positive CHB	HBsAg loss ^d^: 9.7% vs. 0% vs. 5.2%	
		HBeAg-negative CHB	HBsAg loss ^d^: 11.0% vs. 0% vs. 1.3%	
PEG-IFN alfa 2b + NA ^f^ vs. NA ^f^ alone	RCT	HBeAg-positive CHB	HBeAg seroconversion ^g^: 30% vs. 7%	[65]
PEG-IFN alfa 2a + ETV vs. ETV alone	RCT	HBeAg-positive CHB	Liver cirrhosis evaluation by transient elastography value: 6.6 [4.9, 9.8] vs. 7.8 [5.4, 11.1] kPa, *p* = 0.028	[66]
Early combination of PEG-IFN alfa 2a + NA ^h^ vs. NA ^h^	RCT	HBV DNA (+) HCC	8 years OS rate: 79.2% vs. 59.1% 8 years RFS rate: 68.4% vs. 32.3% HBsAg reduced by >1500 IU/mL: 56.5% vs. 41.7%	[67]

Abbreviation: ADV: adefovir dipivoxil; CHB: chronic hepatitis B; ETV: entecavir; HBeAg: hepatitis B e antigen; HBsAg: hepatitis B surface antigen; HBV: hepatitis B virus; HCC: hepatocellular carcinoma; LAM: lamivudine; MA: meta-analysis; NA: nucleos(t)ide analogues; OS: overall survival; RCT: randomised controlled trial; RFS: recurrence-free survival; TDF: tenofovir disoproxil fumarate. Note: ^a^ Virology response: percentage of patients with HBV DNA levels < 2000 IU/mL at the end of treatment. ^b^ Biochemical response: normalization of ALT levels at the end of treatment. ^c^ Percentage of patients with HBeAg seroconversion at the end of treatment. ^d^ Percentage of patients with HbsAg loss at study week 120. ^e^ Percentage of patients with HBeAg seroconversion at study week 120. ^f^ NA: ETV or TDF. ^g^ Percentage of patients with HBeAg seroconversion at study week 96. ^h^ NA: ETV alone or ETV + ADV.

## Data Availability

Not applicable.

## References

[1] Foser S., Schacher A., Weyer K.A., Brugger D., Dietel E., Marti S., Schreitmüller T. (2003). Isolation, structural characterization, and antiviral activity of positional isomers of monopegylated interferon alpha-2a (PEGASYS). Protein Expr. Purif..

[2] Wang Y.-S., Youngster S., Grace M., Bausch J., Bordens R., Wyss D.F. (2002). Structural and biological characterization of pegylated recombinant interferon alpha-2b and its therapeutic implications. Adv. Drug Deliv. Rev..

[3] Huang Y.-W., Qin A., Fang J., Wang T.-F., Tsai C.-W., Lin K.-C., Teng C.-L., Larouche R. (2021). Novel long-acting ropeginterferon alfa-2b: Pharmacokinetics, pharmacodynamics, and safety in a phase 1 clinical trial. Br. J. Clin. Pharmacol..

[4] Huang Y.-W., Tsai C.-Y., Tsai C.-W., Wang W., Zhang J., Qin A., Teng C., Song B., Wang M. (2021). Pharmacokinetics and pharmacodynamics of novel long acting ropeginterferon alfa-2b in healthy Chinese subjects. Adv. Ther..

[5] Miyachi N., Zagrijtschuk O., Kang L., Yonezu K., Qin A. (2021). Pharmacokinetics and pharmacodynamics of ropeginterferon alfa-2b in healthy Japanese and Caucasian subjects after single subcutaneous administration. Clin. Drug Investig..

[6] Gisslinger H., Klade C., Georgiev P., Krochmalczyk D., Gercheva-Kyuchukova L., Egyed M., Rossiev V., Dulicek P., Illes A., Pylypenko H. (2020). Ropeginterferon alfa-2b versus standard therapy for polycythaemia vera (PROUD-PV and CONTINUATION-PV): A randomised, non-inferiority, phase 3 trial and its extension study. Lancet Haematol..

[7] US FDA FDA Approves Treatment for Rare Blood Disease. https://www.fda.gov/news-events/press-announcements/fda-approves-treatment-rare-blood-disease.

[8] Červinek L. (2020). Ropeginterferon alfa-2 b for the therapy of polycythemia vera. Vnitr. Lek..

[9] The National Comprehensive Cancer Network (2022). Guidelines Panel for Myeloproliferative Neoplasms. https://www.nccn.org/guidelines/guidelines-detail?category=1&id=1477.

[10] Lin H.-H., Hsu S.-J., Lu S.-N., Chuang W.-L., Hsu C.-W., Chien R.-N., Yang S.-S., Su W.-W., Wu J.-C., Lee T.-H. (2021). Ropeginterferon alfa-2b in patients with genotype 1 chronic hepatitis C: Pharmacokinetics, safety, and preliminary efficacy. JGH Open.

[11] Hsu S.-J., Yu M.-L., Su C.-W., Peng C.-Y., Chien R.-N., Lin H.-H., Lo G.-H., Su W.-W., Kuo H.-T., Hsu C.-W. (2021). Ropeginterferon alfa-2b administered every two weeks for patients with genotype 2 chronic hepatitis C. J. Formos. Med. Assoc..

[12] Huang Y.-W., Hsu C.-W., Lu S.-N., Yu M.-L., Su C.-W., Su W.-W., Chien R.-N., Hsu C.-S., Hsu S.-J., Lai H.-C. (2020). Ropeginterferon alfa-2b every 2 weeks as a novel pegylated interferon for patients with chronic hepatitis B. Hepatol. Int..

[13] Sebastiani G., Gkouvatsos K., Pantopoulos K. (2014). Chronic hepatitis C and liver fibrosis. World J. Gastroenterol..

[14] El-Serag H.B., Kanwal F. (2014). Epidemiology of hepatocellular carcinoma in the United States: Where are we? Where do we go?. Hepatology.

[15] WHO Hepatitis C Fact Sheets (Updated July 2021). https://www.who.int/news-room/fact-sheets/detail/hepatitis-c.

[16] Gower E., Estes C., Blach S., Razavi-Shearer K., Razavi H. (2014). Global epidemiology and genotype distribution of the hepatitis C virus infection. J. Hepatol..

[17] Smith D.B., Bukh J., Kuiken C., Muerhoff A.S., Rice C.M., Stapleton J.T., Simmonds P. (2014). Expanded classification of hepatitis C virus into 7 genotypes and 67 subtypes: Updated criteria and genotype assignment web resource. Hepatology.

[18] Chung R.T., Gale M., Polyak S.J., Lemon S.M., Liang T.J., Hoofnagle J.H. (2008). Mechanisms of action of interferon and ribavirin in chronic hepatitis C: Summary of a workshop. Hepatology.

[19] Zanin N., Viaris de Lesegno C., Lamaze C., Blouin C.M. (2021). Interferon receptor trafficking and signaling: Journey to the cross roads. Front. Immunol..

[20] Aricò E., Castiello L., Capone I., Gabriele L., Belardelli F. (2019). Type I interferons and cancer: An evolving story demanding novel clinical applications. Cancers.

[21] Lagging M., Langeland N., Pedersen C., Färkkilä M., Buhl M.R., Mørch K., Dhillon A.P., Alsiö A., Hellstrand K., Westin J. (2008). Randomized comparison of 12 or 24 weeks of peginterferon alpha-2a and ribavirin in chronic hepatitis C virus genotype 2/3 infection. Hepatology.

[22] Tsubota A., Satoh K., Aizawa M., Takamatsu S., Namiki Y., Ohkusa T., Fujise K., Tajiri H. (2008). Four-week pegylated interferon alpha-2a monotherapy for chronic hepatitis C with genotype 2 and low viral load: A pilot, randomized study. World J. Gastroenterol..

[23] Toyoda H., Kumada T., Kiriyama S., Sone Y., Tanikawa M., Hisanaga Y., Kanamori A., Atsumi H., Nakano S., Arakawa T. (2009). Eight-week regimen of antiviral combination therapy with peginterferon and ribavirin for patients with chronic hepatitis C virus genotype 2 and a rapid virological response. Liver Int..

[24] Hsu C.-S., Chao Y.-C., Lin H.H., Chen D.-S., Kao J.-H. (2015). Systematic review: Impact of interferon-based therapy on HCV-related hepatocellular carcinoma. Sci. Rep..

[25] Hsu C.-S., Huang C.-J., Kao J.-H., Lin H.H., Chao Y.-C., Fan Y.-C., Tsai P.-S. (2013). Interferon-based therapy decreases risks of hepatocellular carcinoma and complications of cirrhosis in chronic hepatitis C patients. PLoS ONE.

[26] Shen Y.-C., Hsu C., Cheng C.-C., Hu F.-C., Cheng A.-L. (2012). A critical evaluation of the preventive effect of antiviral therapy on the development of hepatocellular carcinoma in patients with chronic hepatitis C or B: A novel approach by using meta-regression. Oncology.

[27] Merck Sharp & Dohme Corp. (2019). Package Insert: PEGINTRON® (Peginterferon alfa-2b).

[28] Roche (2002). Package Insert: PEGASYS® (Peginterferon alfa-2a).

[29] WHO Hepatitis B Fact Sheets (Updated July 2021). https://www.who.int/news-room/fact-sheets/detail/hepatitis-b.

[30] Tseng T.-C., Kao J.-H. (2017). Elimination of hepatitis B: Is it a mission possible?. BMC Med..

[31] Alexopoulou A., Vasilieva L., Karayiannis P. (2020). New approaches to the treatment of chronic hepatitis B. J. Clin. Med..

[32] Suk-Fong Lok A. (2019). Hepatitis B treatment: What we know now and what remains to be researched. Hepatol. Commun..

[33] Di Stefano M., Faleo G., Farhan Mohamed A.M., Morella S., Bruno S.R., Tundo P., Fiore J.R., Santantonio T.A. (2021). Resistance associated mutations in HCV patients failing DAA treatment. New Microbiol..

[34] Starace M., Minichini C., De Pascalis S., Macera M., Occhiello L., Messina V., Sangiovanni V., Adinolfi L.E., Claar E., Precone D. (2018). Virological patterns of HCV patients with failure to interferon-free regimens. J. Med. Virol..

[35] Sarrazin C. (2021). Treatment failure with DAA therapy: Importance of resistance. J. Hepatol..

[36] Malandris K., Kalopitas G., Theocharidou E., Germanidis G. (2021). The role of RASs/RVs in the current management of HCV. Viruses.

[37] Reig M., Mariño Z., Perelló C., Iñarrairaegui M., Ribeiro A., Lens S., Díaz A., Vilana R., Darnell A., Varela M. (2016). Unexpected high rate of early tumor recurrence in patients with HCV-related HCC undergoing interferon-free therapy. J. Hepatol..

[38] Conti F., Buonfiglioli F., Scuteri A., Crespi C., Bolondi L., Caraceni P., Foschi F.G., Lenzi M., Mazzella G., Verucchi G. (2016). Early occurrence and recurrence of hepatocellular carcinoma in HCV-related cirrhosis treated with direct-acting antivirals. J. Hepatol..

[39] Cardoso H., Vale A.M., Rodrigues S., Gonçalves R., Albuquerque A., Pereira P., Lopes S., Silva M., Andrade P., Morais R. (2016). High incidence of hepatocellular carcinoma following successful interferon-free antiviral therapy for hepatitis C associated cirrhosis. J. Hepatol..

[40] Urbanowicz A., Zagożdżon R., Ciszek M. (2019). Modulation of the immune system in chronic hepatitis C and during antiviral interferon-free therapy. Arch. Immunol. Ther. Exp..

[41] Oe N., Takeda H., Eso Y., Takai A., Marusawa H. (2022). Clinical and molecular basis of hepatocellular carcinoma after hepatitis C virus eradication. Pathogens.

[42] Qin X.-Q., Runkel L., Deck C., DeDios C., Barsoum J. (1997). Interferon-beta induces S phase accumulation selectively in human transformed cells. J. Interferon Cytokine Res..

[43] Qin X.-Q., Beckham C., Brown J.L., Lukashev M., Barsoum J. (2001). Human and mouse IFN-β gene therapy exhibits different anti-tumor mechanisms in mouse models. Mol. Ther..

[44] Kaynor C., Xin M., Wakefield J., Barsoum J., Qin X.-Q. (2002). Direct evidence that IFN-beta functions as a tumor-suppressor protein. J. Interferon Cytokine Res..

[45] Brickelmaier M., Carmillo A., Goelz S., Barsoum J., Qin X.-Q. (2002). Cytotoxicity of combinations of IFN-beta and chemotherapeutic drugs. J. Interferon Cytokine Res..

[46] Qin X.-Q., Tao N., Dergay A., Moy P., Fawell S., Davis A., Wilson J.M., Barsoum J. (1998). Interferon-beta gene therapy inhibits tumor formation and causes regression of established tumors in immune-deficient mice. Proc. Natl. Acad. Sci. USA.

[47] Hoffmann-La Roche, Inc. (2008). Package Insert: ROFERON®—A (Interferon alfa-2a, Recombinant).

[48] Sondak V.K., Kudchadkar R. (2012). Pegylated interferon for the adjuvant treatment of melanoma: FDA approved, but what is its role?. Oncologist.

[49] MERCK & Co., Inc. (2021). Package Insert: SYLATRON^TM^ (Peginterferon alfa-2b).

[50] Magenau J.M., Pawarode A., Riwes M.M., Parkin B., Anand S., Ghosh M., Bixby D.L., Choi S., Bischoff L., Yanik G.A. (2019). A Phase I/II clinical trial of type 1 interferon for reduction of relapse after HCT in high risk AML. Biol. Blood Marrow Transplant..

[51] Mishra P., Nayak B., Dey R.K. (2016). PEGylation in anti-cancer therapy: An overview. Asian J. Pharm. Sci..

[52] Hjorth-Hansen H., Stentoft J., Richter J., Koskenvesa P., Höglund M., Dreimane A., Porkka K., Gedde-Dahl T., Gjertsen B.T., Gruber F.X. (2016). Safety and efficacy of the combination of pegylated interferon-α2b and dasatinib in newly diagnosed chronic-phase chronic myeloid leukemia patients. Leukemia.

[53] Healy F.M., Dahal L.N., Jones J.R.E., Floisand Y., Woolley J.F. (2021). Recent progress in interferon therapy for myeloid malignancies. Front. Oncol..

[54] Hung H.-C., Liao H.-H., Chen S.-C., Tsao S.-M., Lee Y.-T. (2019). Maintenance interferon therapy in chronic hepatitis C patients who failed initial antiviral therapy: A meta-analysis. Medicine.

[55] Ma L., Liu J., Wang W., Yang F., Li P., Cai S., Zhou X., Chen X., Zhuang X., Zhang H. (2020). Direct-acting antivirals and interferon-based therapy on hepatocellular carcinoma risk in chronic hepatitis-C patients. Future Oncol..

[56] Gisslinger H., Zagrijtschuk O., Buxhofer-Ausch V., Thaler J., Schloegl E., Gastl G.A., Wolf D., Kralovics R., Gisslinger B., Strecker K. (2015). Ropeginterferon alfa-2b, a novel IFNα-2b, induces high response rates with low toxicity in patients with polycythemia vera. Blood.

[57] Verger E., Soret-Dulphy J., Maslah N., Roy L., Rey J., Ghrieb Z., Kralovics R., Gisslinger H., Grohmann-Izay B., Klade C. (2018). Ropeginterferon alpha-2b targets JAK2V617F-positive polycythemia vera cells in vitro and in vivo. Blood Cancer J..

[58] Alavian S.-M., Tabatabaei S.V., Behnava B., Rizzetto M. (2012). Standard and pegylated interferon therapy of HDV infection: A systematic review and meta- analysis. J. Res. Med. Sci..

[59] Wang G., Guan J., Khan N.U., Li G., Shao J., Zhou Q., Xu L., Huang C., Deng J., Zhu H. (2021). Potential capacity of interferon-α to eliminate covalently closed circular DNA (cccDNA) in hepatocytes infected with hepatitis B virus. Gut Pathog..

[60] Lai C.-L., Wong D., Ip P., Kopaniszen M., Seto W.-K., Fung J., Huang F.-Y., Lee B., Cullaro G., Chong C.K. (2017). Reduction of covalently closed circular DNA with long-term nucleos(t)ide analogue treatment in chronic hepatitis B. J. Hepatol..

[61] Tamai H., Ida Y., Shingaki N., Shimizu R., Fukatsu K., Itonaga M., Yoshida T., Maeda Y., Moribata K., Maekita T. (2017). Add-on pegylated interferon alpha-2a therapy in chronic hepatitis B Japanese patients treated with entecavir. Hepat. Res. Treat..

[62] Chen G.-Y., Su T.-H., Kao J.-H. (2015). Successful treatment of chronic hepatitis B and D with pegylated-interferon plus entecavir. J. Formos. Med. Assoc..

[63] Kim V., Abreu R.M., Nakagawa D.M., Baldassare R.M., Carrilho F.J., Ono S.K. (2016). Pegylated interferon alfa for chronic hepatitis B: Systematic review and meta-analysis. J. Viral Hepat..

[64] Ahn S.H., Marcellin P., Ma X., Caruntu F.A., Tak W.Y., Elkhashab M., Chuang W.-L., Tabak F., Mehta R., Petersen J. (2018). Hepatitis B surface antigen loss with tenofovir disoproxil fumarate plus peginterferon alfa-2a: Week 120 analysis. Dig. Dis. Sci..

[65] Chi H., Hansen B.E., Guo S., Zhang N.P., Qi X., Chen L., Guo Q., Arends P., Wang J.-Y., Verhey E. (2017). Pegylated interferon alfa-2b add-on treatment in hepatitis B virus envelope antigen-positive chronic hepatitis B patients treated with nucleos(t)ide analogue: A randomized, controlled trial (PEGON). J. Infect. Dis..

[66] Yang J.-M., Chen L.-P., Wang Y.-J., Lyu B., Zhao H., Shang Z.-Y., Li J., Fan Z.-Y., Wu S.-D., Ming X. (2020). Entecavir add-on Peg-interferon therapy plays a positive role in reversing hepatic fibrosis in treatment-naïve chronic hepatitis B patients: A prospective and randomized controlled trial. Chin. Med. J..

[67] Qi W., Zhang Q., Xu Y., Wang X., Yu F., Zhang Y., Zhao P., Guo H., Zhou C., Wang Z. (2020). Peg-interferon and nucleos(t)ide analogue combination at inception of antiviral therapy improves both anti-HBV efficacy and long-term survival among HBV DNA-positive hepatocellular carcinoma patients after hepatectomy/ablation. J. Viral Hepat..

[68] WHO Hepatitis D Fact Sheets (Updated July 2021). https://www.who.int/news-room/fact-sheets/detail/hepatitis-d.

[69] Papatheodoridi M., Papatheodoridis G.V. (2021). Current status of hepatitis delta. Curr. Opin. Pharmacol..

[70] Urban S., Neumann-Haefelin C., Lampertico P. (2021). Hepatitis D virus in 2021: Virology, immunology and new treatment approaches for a difficult-to-treat disease. Gut.

[71] Rizzetto M., Hamid S., Negro F. (2021). The changing context of hepatitis D. J. Hepatol..

[72] Alfaiate D., Clément S., Gomes D., Goossens N., Negro F. (2020). Chronic hepatitis D and hepatocellular carcinoma: A systematic review and meta-analysis of observational studies. J. Hepatol..

[73] Terrault N.A., Lok A.S.F., McMahon B.J., Chang K.-M., Hwang J.P., Jonas M.M., Brown R.S., Bzowej N.H., Wong J.B. (2018). Update on prevention, diagnosis, and treatment of chronic hepatitis B: AASLD 2018 hepatitis B guidance. Hepatology.

[74] Sagnelli C., Sagnelli E., Russo A., Pisaturo M., Occhiello L., Coppola N. (2021). HBV/HDV co-Infection: Epidemiological and clinical changes, recent knowledge and future challenges. Life.

[75] Wedemeyer H., Yurdaydìn C., Dalekos G.N., Erhardt A., Çakaloğlu Y., Değertekin H., Gürel S., Zeuzem S., Zachou K., Bozkaya H. (2011). Peginterferon plus adefovir versus either drug alone for hepatitis delta. N. Engl. J. Med..

[76] Farci P., Roskams T., Chessa L., Peddis G., Mazzoleni A.P., Scioscia R., Serra G., Lai M.E., Loy M., Caruso L. (2004). Long-term benefit of interferon alpha therapy of chronic hepatitis D: Regression of advanced hepatic fibrosis. Gastroenterology.

[77] Bazinet M., Pântea V., Cebotarescu V., Cojuhari L., Jimbei P., Anderson M., Gersch J., Holzmayer V., Elsner C., Krawczyk A. (2021). Persistent control of hepatitis B virus and hepatitis delta virus infection following REP 2139-Ca and pegylated interferon therapy in chronic hepatitis B virus/hepatitis delta virus coinfection. Hepatol. Commun..

[78] Wranke A., Hardtke S., Heidrich B., Dalekos G., Yalçin K., Tabak F., Gürel S., Çakaloğlu Y., Akarca U.S., Lammert F. (2020). Ten-year follow-up of a randomized controlled clinical trial in chronic hepatitis delta. J. Viral Hepat..

[79] Heidrich B., Yurdaydın C., Kabaçam G., Ratsch B.A., Zachou K., Bremer B., Dalekos G.N., Erhardt A., Tabak F., Yalcin K. (2014). Late HDV RNA relapse after peginterferon alpha-based therapy of chronic hepatitis delta. Hepatology.

[80] Kang C., Syed Y.Y. (2020). Bulevirtide: First approval. Drugs.

[81] European Medicines Agency (2020). Hepcludex-EPAR-Assessment-Report_en. https://www.ema.europa.eu/en/medicines/human/EPAR/hepcludex.

[82] Hepatera Ltd. Study Results of ClinicalTrials.gov No. NCT03546621, Last Updated 10 May 2021. NCT03546621.

[83] Hepatera Ltd. Study Results of ClinicalTrials.gov No. NCT02888106, Last Updated 30 April 2021. NCT02888106.

[84] Yurdaydin C., Keskin O., Yurdcu E., Çalişkan A., Önem S., Karakaya F., Kalkan Ç., Karatayli E., Karatayli S., Choong I. (2021). A phase 2 dose-finding study of lonafarnib and ritonavir with or without interferon alpha for chronic delta hepatitis. Hepatology.

[85] Matsumura T., Hu Z., Kato T., Dreux M., Zhang Y.-Y., Imamura M., Hiraga N., Juteau J.-M., Cosset F.-L., Chayama K. (2009). Amphipathic DNA polymers inhibit hepatitis C virus infection by blocking viral entry. Gastroenterology.

[86] Vaillant A., Juteau J.-M., Lu H., Liu S., Lackman-Smith C., Ptak R., Jiang S. (2006). Phosphorothioate oligonucleotides inhibit human immunodeficiency virus type 1 fusion by blocking gp41 core formation. Antimicrob. Agents Chemother..

[87] Cardin R.D., Bravo F.J., Sewell A.P., Cummins J., Flamand L., Juteau J.-M., Bernstein D.I., Vaillant A. (2009). Amphipathic DNA polymers exhibit antiviral activity against systemic murine cytomegalovirus infection. Virol. J..

[88] Guzman E.M., Cheshenko N., Shende V., Keller M.J., Goyette N., Juteau J.-M., Boivin G., Vaillant A., Herold B.C. (2007). Amphipathic DNA polymers are candidate vaginal microbicides and block herpes simplex virus binding, entry and viral gene expression. Antivir. Ther..

[89] Beilstein F., Blanchet M., Vaillant A., Sureau C. (2018). Nucleic acid polymers are active against hepatitis delta virus infection in vitro. J. Virol..

[90] Noordeen F., Vaillant A., Jilbert A.R. (2013). Nucleic acid polymers prevent the establishment of duck hepatitis B virus infection in vivo. Antimicrob. Agents Chemother..

[91] Al-Mahtab M., Bazinet M., Vaillant A. (2016). Safety and efficacy of nucleic acid polymers in monotherapy and combined with immunotherapy in treatment-naive bangladeshi patients with HBeAg+ chronic hepatitis B infection. PLoS ONE.

[92] Bazinet M., Pântea V., Cebotarescu V., Cojuhari L., Jimbei P., Albrecht J., Schmid P., LeGal F., Gordien E., Krawczyk A. (2017). Safety and efficacy of REP 2139 and pegylated interferon alfa-2a for treatment-naive patients with chronic hepatitis B virus and hepatitis D virus co-infection (REP 301 and REP 301-LTF): A non-randomised, open-label, phase 2 trial. Lancet Gastroenterol. Hepatol..

[93] Carolina B., Paola F., Caterina V., Barbara A., Paola D.V., Tiziana G., Diletta L., Alessandro Z., Albertina C., Gabriele M. (2007). Characterization of hepatitis B virus (HBV)-specific T-cell dysfunction in chronic HBV infection. J. Virol..

[94] Feng C., Cao L.-J., Song H.-F., Xu P., Chen H., Xu J.-C., Zhu X.-Y., Zhang X.-G., Wang X.-F. (2015). Expression of PD-L1 on CD4^+^CD25^+^Foxp3^+^ regulatory T Cells of patients with chronic HBV infection and its correlation with clinical parameters. Viral Immunol..

[95] Tzeng H.-T., Tsai H.-F., Liao H.-J., Lin Y.-J., Chen L., Chen P.-J., Hsu P.-N. (2012). PD-1 blockage reverses immune dysfunction and hepatitis B viral persistence in a mouse animal model. PLoS ONE.

[96] Liu J., Zhang E., Ma Z., Wu W., Kosinska A., Zhang X., Möller I., Seiz P., Glebe D., Wang B. (2014). Enhancing virus-specific immunity in vivo by combining therapeutic vaccination and PD-L1 blockade in chronic hepadnaviral infection. PLoS Pathog..

[97] El-Khoueiry A.B., Sangro B., Yau T., Crocenzi T.S., Kudo M., Hsu C., Kim T.-Y., Choo S.-P., Trojan J., Welling T.H.R. (2017). Nivolumab in patients with advanced hepatocellular carcinoma (CheckMate 040): An open-label, non-comparative, phase 1/2 dose escalation and expansion trial. Lancet.

[98] Gane E., Verdon D.J., Brooks A.E., Gaggar A., Nguyen A.H., Subramanian G.M., Schwabe C., Dunbar P.R. (2019). Anti-PD-1 blockade with nivolumab with and without therapeutic vaccination for virally suppressed chronic hepatitis B: A pilot study. J. Hepatol..

[99] Pu D., Yin L., Zhou Y., Li W., Huang L., Cai L., Zhou Q. (2020). Safety and efficacy of immune checkpoint inhibitors in patients with HBV/HCV infection and advanced-stage cancer: A systematic review. Medicine.

[100] Burns E.A., Muhsen I.N., Anand K., Xu J., Umoru G., Arain A.N., Abdelrahim M. (2021). Hepatitis B virus reactivation in cancer patients treated with immune checkpoint inhibitors. J. Immunother..

[101] Féray C., López-Labrador F.X. (2019). Is PD-1 blockade a potential therapy for HBV?. JHEP Rep..

[102] Brown J.L., Barsoum J., Qin X.-Q. (2002). CD4+ T helper cell-independent antitumor response mediated by murine IFN-beta gene delivery in immunocompetent mice. J. Interferon Cytokine Res..

